# Ferroelectromagnetic Properties of PbFe_1/2_Nb_1/2_O_3_ (PFN) Material Synthesized by Chemical-Wet Technology

**DOI:** 10.3390/ma11122504

**Published:** 2018-12-09

**Authors:** Dariusz Bochenek, Przemysław Niemiec

**Affiliations:** Institute of Technology and Mechatronics, Faculty of Computer Science and Material Science, University of Silesia in Katowice, 12 Zytnia St., 41–200 Sosnowiec, Poland; przemyslaw.niemiec@us.edu.pl

**Keywords:** multifereroics, ferroelectromagnetics, perovskite type materials, chemical-wet technology, sol-gel

## Abstract

In this work, PbFe_1/2_Nb_1/2_O_3_ (PFN) ceramic samples synthesized by chemically wet method (precipitation from the solution) were obtained. Due to the tendency to form powder agglomerates, the synthesized powder was subjected to ultrasound. The sintering was carried out under various technological conditions, mainly through controlling the sintering temperature. -X-ray powder-diffraction (XRD), scanning electron microscope (SEM) microstructure analysis, as well as the examinations of dielectric, ferroelectric, and magnetic properties of the PFN ceramics were carried out. Studies have shown that hard ceramic agglomerates can be partially minimized by ultrasound. Due to this treatment, closed porosity decreases, and the ceramic samples have a higher density. Optimization and improvement of the technological process of the PFN material extends the possibility of its use for the preparation of multiferroic composites or multicomponent solid solutions based on PFN. Such materials with functional properties find applications in microelectronic applications, e.g., in systems integrating ferroelectric and magnetic properties in one device. The optimal synthesis conditions of PFN ceramics were determined to be 1050 °C/2 h.

## 1. Introduction

Ferroelectromagnetic PbFe_1−*x*_Nb*_x_*O_3_ (PFN) material is a member of the perovskite-like family of the general formula A(B′B″)O_3_, exhibiting multiferroic properties. Ions of lead locate to A position, while ions of niobium and iron are substituted randomly to octahedral positions B′ and B″ [1,2]. Multiferroics are materials which show simultaneously at least two types of physical (ferroic) states e.g., a ferromagnetic state (antiferromagnetic, ferrimagnetic) [3], a ferroelectric state (antiferroelectric, ferrielectric), a ferroelastic state (ferromagneticelastic, ferroelastoelectric), and a ferrotoroid state [4,5,6,7,8,9]. In the PFN materials, iron has a natural magnetic moment (connecting Fe-O-Fe in the angle of 180° ensures an optimum state of magnetic order occurrence [10]). Smart materials are used extensively in automation, control processes, robotics, micromechatronic applications, material processing, aerospace engineering, the automotive and electronic industries, defence technology, medical technology and biotechnology [11,12,13,14,15]. On the other hand, the piezoelectric transduction mechanism is also an efficient mechanism for nanoelectronics and microelectronics [16,17].

During the technological process of the PFN ceramic material, many methods and techniques are used, e.g., a sol-gel synthesis, molten salt synthesis, precipitation from the solution, reaction in solid state, two-stage columbite method, and mechanical activation (high-energy milling) [18,19,20,21,22,23,24,25,26]. Each of the above-mentioned methods and techniques require the use of specific technological conditions and diligence in laboratory process in order to obtain materials with good properties. Precipitating nanoparticles from the solution of chemical compounds can be classified into five categories: colloidal methods (precipitation processes), sol–gel processing, water–oil micro-emulsions method, hydrothermal synthesis, and polyol method. The main benefits of liquid phase synthesis are a high purity and uniform nanostructure achievable at low temperatures. Generally, the sol-gel process consists in the chemical transformation of a liquid (sol) into a gel state (in various forms), and subsequently, a transition into solid powders (during further processing). The sol-gel method, and the numerous varieties thereof, allowing one to obtain a ceramic powder with optimal parameters at low synthesis temperatures, which provides preservation of the stoichiometric composition. The sol-gel synthesis may be used to prepare materials with different shapes, i.e., dense or porous structures, thin fibres, thin films etc. [27,28,29,30,31]. The advantages of the solution–precipitation method in the technological process of the synthesis of ceramic materials are a high purity and a uniform nanostructure achievable at low temperatures. PFN ceramics can be used in modern microelectric and micromechatronic applications, such as piezoelectric transducers, sensors, generators, servomotors, actuators, phase modulators, frequency multipliers, multilayer ceramic capacitors (MLCC), and memory elements [2,32].

It has already been shown that single-phase PFN materials reveal weak magnetoelectric coupling [33,34]. The improvement of the known technological methods and the optimization of the technological process allows one to obtain a PFN material with repetitive properties and optimal operational parameters. In order to further improve the electrophysical properties and increase the application functionality, the PbFe_1/2_Nb_1/2_O_3_ material can be used, for instance, to obtain multicomponent solid solutions [34,35,36,37,38], to further modify the basic composition with appropriate admixtures [39,40,41], or as an element of ceramic composite ferroelectromagnetics [42] in micromechatronic applications, integrating various properties in one system.

The aim of the work is to obtain and study the ferroelectromagnetic PFN ceramics synthesized by a chemical-wet technology (solution precipitation method) and sintered by conventional pressureless methods. As a result of the technological process used, the PFN ceramic powder tended to combine into large, hard agglomerates. In order to minimize this adverse phenomenon, at the initial stage of the process, the powder agglomerates were subjected to ultrasound disruption.

The crystal structure, microstructure, DC electric conductivity, magnetic, dielectric, and ferroelectric properties of the PFN samples were investigated.

## 2. Experiment

**Figure 1:**
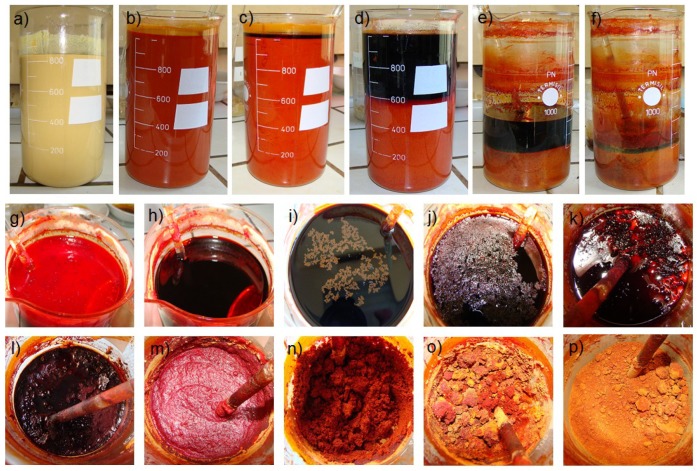
The precipitation method of the PbFe_1/2_Nb_1/2_O_3_ material: (**a**–**f**) side view, (**g**–**p**) top view. Successive stages of the technological process: (**a**) mixing ingredients, (**b**,**g**) introducing acetylacetone and distilled water, (**c**–**f**) and (**h**–**l**) the solvent vaporization (as a result of the IR lamp effect on the solution), (**m**–**o**) powder formation, (**p**) powder after completion of drying.

The synthesized ceramic PbFe_1−*x*_Nb*_x_*O_3_ (PFN) powder was obtained by a chemical-wet technology—the solution precipitation method [24] (a variation of the sol-gel method). In the technological process (Figure 1), there were the following precursors: ferric citrate monohydrate C_6_H_5_FeO_7_⋅H_2_O (pure p.a., Fluka, Neu-Ulm, Germany), lead (II) acetate trihydrate Pb(CH_3_COO)_2_⋅3H_2_O (pure p.a., POCH), and niobium (V) ethoxide Nb(OC_2_H_5_)_5_ (99.95%, Aldrich, Saint Louis, MO, USA). Lead acetate trihydrate was dissolved in the CH_3_COOH acetic acid, and simultaneously Nb(OC_2_H_5_)_5_ ethylene glycol (POCH) with 2-methoxyethanol (Fluka) was added. Subsequently, distilled water was added to ferric citrate monohydrate (the solution was heated). Next, acetic acid was added to increase the effectiveness of dissolving and it was brought to boiling (with vigorous stirring). Consecutively, all of the ingredients were mixed together and acetylacetone was added, with the entire mixture being heated simultaneously. In the next stage, distilled water was added for the hydrolysis process and the precipitation process started. The obtained liquid solution was dried under an IR lamp (allowing evaporation of the solvent to occur faster, producing a ceramic powder in the final stage). After completion of drying the powder was mixed, after which the removal of organic parts was done by calcination route at 600 °C/3 h. As a result of the technological process, fine-grained a ceramic powder was obtained.

The obtained fine-grained ceramic powder of PFN, during the technological process, tended to form numerous agglomerates, which promoted the formation of a significant closed porosity in the volume of ceramic samples [24]. Since the optimization of powder mixing parameters used during the experiment as well as the extension of the mixing time did not eliminate the tendency to form hard, strongly compact powder agglomerates, an additional step in powder technology was applied. In order to minimize this unfavorable phenomenon, the process of breaking up agglomerates with ultrasound was carried out in an ultrasonic cleaner.

In the subsequent stage, the PFN powder was mixed and then pressed into pellets. Densification was made by the free sintering method (obtaining three ceramic samples) at the following conditions (*T_s_*/*t_s_*): (i) 1025 °C/2 h (P-1u), (ii) 1050 °C/2 h (P-2u), (iii) 1075 °C/2 h (P-3u). As the obtained PFN ceramic samples have closed porosity, the ceramic powder was again mixed in a ball mill for 24 h, and then the powder was twice calcined at 600 °C/2 h. After these treatments, the ceramic powder was pressed into pellets and densification was conducted by the free sintered method, at the following conditions: (iv) 1050 °C/2 h (P-4u).

For the electrical measurements, sintered pellets with a thickness of 1.0 mm were prepared. The mechanical stress was removed by the annealing process (750 °C/0.25 h), and surfaces of the samples were covered with a silver electrode at 850 °C/0.25 h (method of firing a silver paste).

X-ray powder-diffraction data (XRD) of the PFN material were performed on a Phillips X’Pert APD diffractometer (PANalytical, Eindhoven, The Netherlands) at room temperature (Cu-Kα radiation, in the 2*θ* range from 10° to 65°, in steps of 0.02°, with an integration time of 4 s/step). The surface morphology and stoichiometry of the PFN ceramics were tested on a JSM-7100F TTL LV (Jeol Lid., Tokyo, Japan) scanning electron microscope (the samples were coated with gold to provide electrical conductivity in order to obtain charging effects). Temperature dielectric measurements were performed on a LCR meter (QuadTech 1920 Precision LCR Meter, QuadTech, Inc., Maynard, MA, USA) and DC electrical conductivity was carried out using a Keithley 6517B electrometer (Keithley Instruments, Cleveland, OH, USA), in a temperature range from 20 °C to 420 °C (a heating cycle). 

Ferroelectric properties were carried out using a Sawyer-Tower circuit and a high voltage amplifier (Matsusada Inc. HEOPS-5B6 precision, Matsusada Precision Inc., Kusatsu, Japan). The experimental data were stored on a computer disc using an A/D, D/A transducer card (National Instruments Corporation, Austin, TX, USA). The DC magnetic susceptibility measurements were carried out by the Cahn magnetic balance (*H*_0_ = 800 Oe).

## 3. Results and Discussion

### 3.1. X-Ray Diffraction Analysis

**Figure 2:**
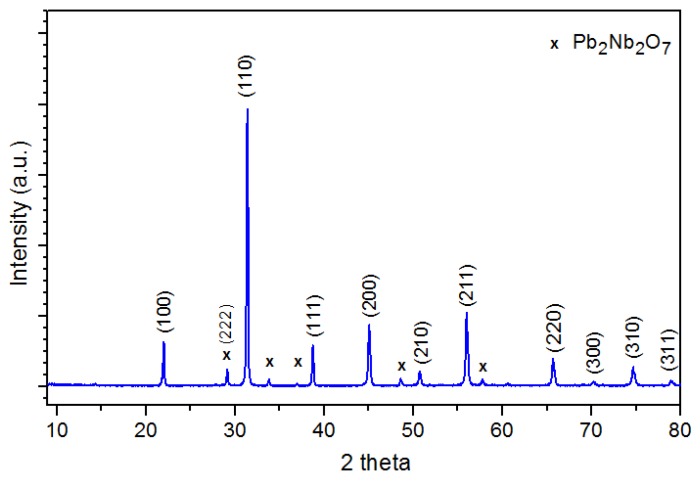
X-ray diffraction patterns of the Pb_2_Nb_2_O_7_ (PFN) materials.

At room temperature, the PFN ceramic material obtained by the solution precipitation method has shown a perovskite structure with a small amount of the pyrochlore phase (Pb_2_Nb_2_O_7_). The best match results (Figure 2) were obtained for the pattern (JCPDS card No. 04-009-5124), with tetragonal symmetry and P*4mm* space groups (the unit cell parameters: *a*_0_ = 4.0070 Å, *b*_0_ = 4.0070 Å, *c*_0_ = 4.0130 Å, and *α* = 90°). From the integrated intensities of the fitted peaks, the perovskite phase in the ceramic PFN powder was estimated using the Equation (1)

(1) $$P_{p}=\frac{I_{110}\cdot 100}{I_{110}+I_{222}}\;\left[\%\right]$$

where *I*_110_ and *I*_222_ are integrated intensities of the most intense (110) perovskite and (222) pyrochlore diffraction lines. The percentage content of the perovskite phase in the PFN ceramic material is 94.64.

### 3.2. Microstructural Properties

**Figure 3:**
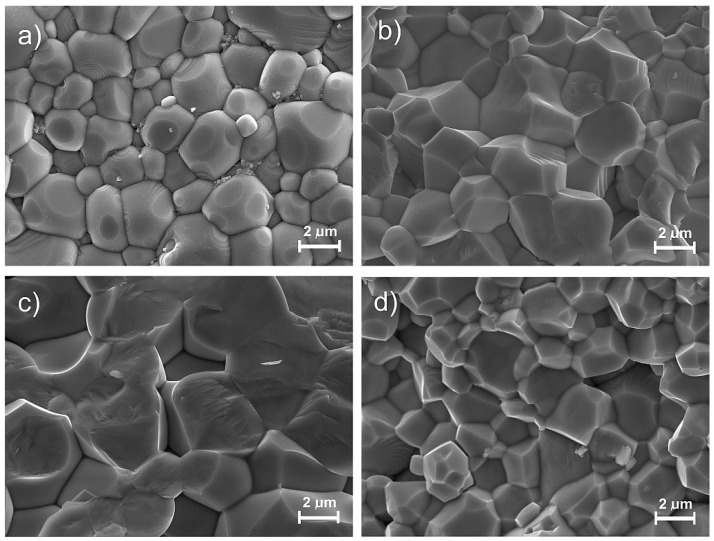
SEM (scanning electron microscope) images of the PFN ceramic samples: (**a**) P-1u, (**b**) P-2u, (**c**) P-3u, (**d**) P-4u.

The surface morphology of the fracture PFN samples with different technologies is shown in Figure 3. The microstructure of the P-1u sample shows grain size heterogeneity with clearly visible grain boundaries. In the microstructure, there are both small and large grains with incomplete crystallization. In the case of the P-2u and P-4u samples, the microstructure is characterized by properly crystallized grains, but with visible porosity. The sample breakthrough occurs mainly through grain boundaries, which is confirmed by the properly selected technological sintering conditions of the ceramic, ensuring high mechanical strength of the grain interior. In the case of the P-3u sample, additional stages of the technological process have caused higher grains in the microstructure. The breakthrough of the sample takes place predominantly through the grain, however breaks occurs in a compact solid manner. This behavior indicates high mechanical strength in the grain boundaries, at the expense of the interior of the grains. Activation of ultrasound on the ceramic powder of PFN, after the synthesis process, partially breaks hard powder agglomerates. As a result, the PFN ceramics have a higher density, with a smaller amount of closed porosity.

**Figure 4:**
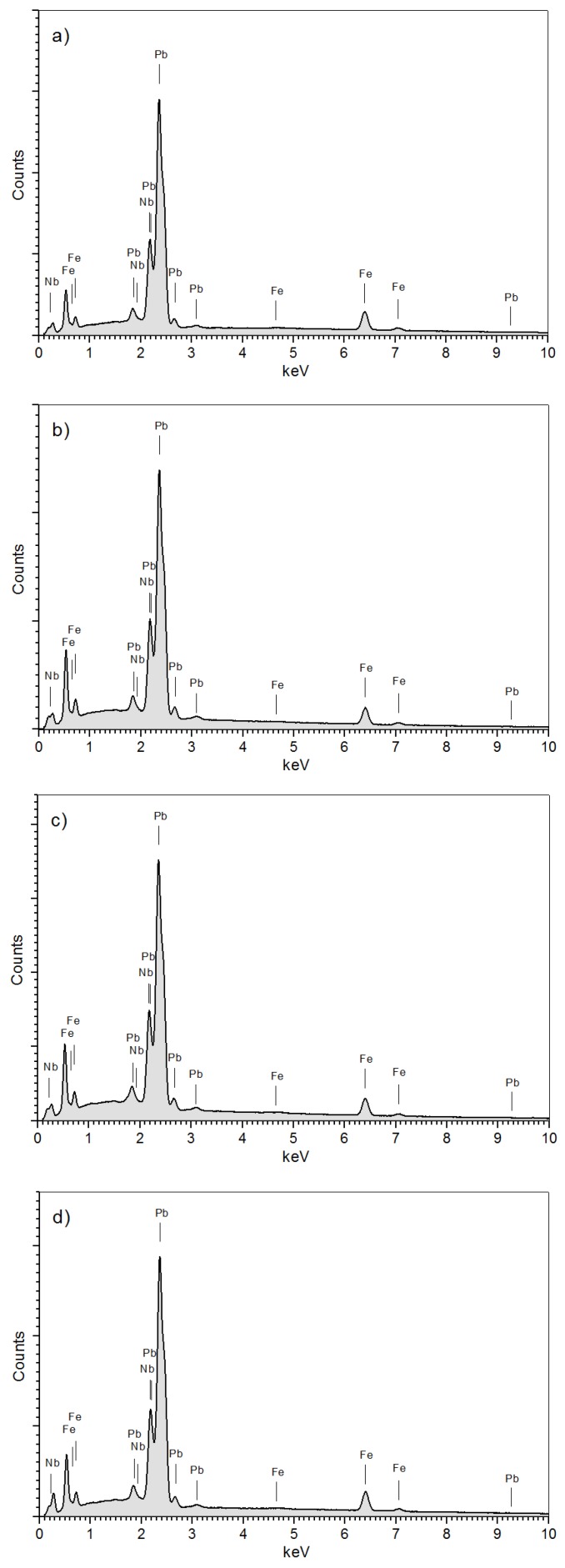
Energy dispersive spectrometry (EDS) testes of the PFN samples: (**a**) P-1u, (**b**) P-2u, (**c**) P-3u, (**d**) P-4u.

The energy dispersive spectrometry (EDS) analysis (average of ten randomly chosen areas from the specimen surface) of the PFN ceramic samples confirmed the presence of constituent elements in the obtained materials. In Figure 4, all analyzed PFN samples have characteristic peaks, derived from constituent elements, differing slightly in the intensity of reflections (without the presence of peaks coming from other elements). The calculated percentage of the individual components of the PFN ceramics was summarized in Table 1. The EDS analysis of the element distribution revealed some differences in the chemical composition. All deviations between the theoretical content of chemical elements and their real content are within an acceptable range. In the case of the P-1u and P-2u samples, iron and lead deficiency and a small excess of niobium are observed, while in the case of the P-3u and P-4u samples there is a small excess of iron. The highest percentage deviation from the theoretical composition is for the P-1u samples sintered at the lowest temperature. The EDS research confirmed that the technological process of the PFN ceramics was performed correctly (no significant deviations from the assumed composition).

### 3.3. DC Electrical Conductivity Measurements

**Figure 5:**
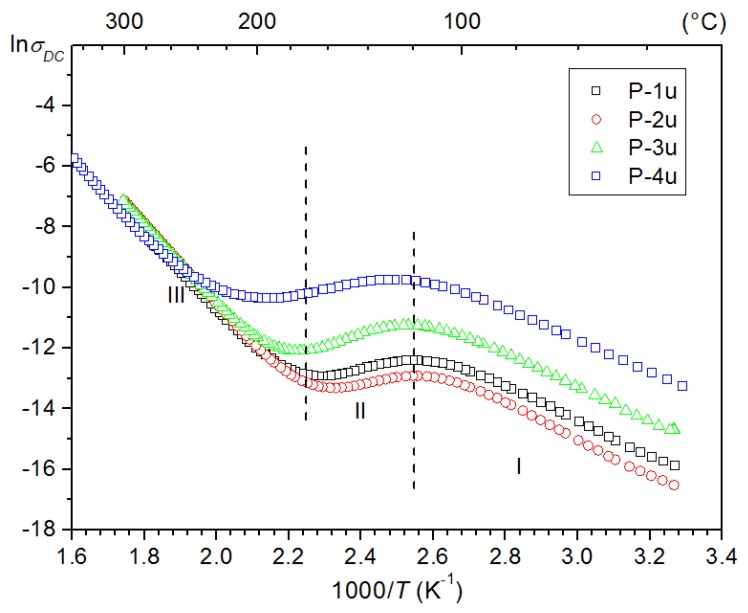
The ln*σ_DC_* (1000/*T*) relationship for the PFN ceramics.

The *ρ_DC_* resistivity at room temperature of the PFN samples is typical for semiconductors and is summarized in Table 1. The highest resistivity is revealed by the P-2u sample (1.4 × 10^7^ Ω m), while smaller is for the P-4u one (6.1 × 10^5^ Ω m). In the case of the P-4u ceramic samples, additional stages of the technological process led to increased electric conductivity (Figure 5). It is may be connected both with lower resistance of the ceramic grains, as well as grain boundaries (i.e., a temperature effect of synthesis associated with irreversible chemical changes on the grain boundaries [43]). The PFN ceramic materials exhibit *n*-type conductivity, which may be determined by the oxygen deficiency [44]. The oxygen deficiency results in the formation of oxygen vacancies in the crystal lattice (donor centres because of ionisation processes). The reduction processes may lead to the creation of oxygen vacancies or valency change in Nb^5+^ and Fe^3+^ ions and the formation of lattice defects (i.e., *V_O_*, $V_{O}^{•}$, $Fe_{Fe}^{\prime }$, $Nb_{Nb}^{\prime }$) and complexes $V_{O}^{•}Fe_{Fe}^{\prime }$ and $V_{O}^{•}Nb_{Nb}^{\prime }$, which can be donor centers. Nonstoichiometry in the oxygen sublattice can be a result of reduction processes proceeding at high temperature, during technological process of the perovskite-type materials [44].

Activation energy for PFN ceramic samples was calculated according to Arrhenius’ law (Equation (2)) [45]:

(2) $$\sigma =\sigma_{0}exp\left(\frac{E_{Act}}{k_{B}T}\right)$$

where *σ*_0_ is pre-exponential factor, *k_B_* is Boltzmann’s constant, *T* is absolute temperature, and *E_Act_* is the activation energy appointed from the slope of ln*σ_DC_* vs. 1/*T* plot. The values of the activation energy *E_Act_* at three specific regions (selected in Figure 5) are summarized in Table 2. Activation energy of the conductivity in the first region is rather small (from 0.146 eV for P-2u sample to 3.02 eV for P-3u sample). In this region, the activation energy is mostly connected with charge carrier mobility. It is shown on a hopping mechanism of the conductivity associated with the occurence of oxygen vacancies and valency change of transition elements Fe and Nb [46].

Like most materials with perovskite structure, in the PFN ceramics at lower temperatures (I area—below *T_m_*) the values of activation energy are lower than above the phase transition temperature (III area) (Table 2) [47].

### 3.4. Dielectric Properties

**Figure 6:**
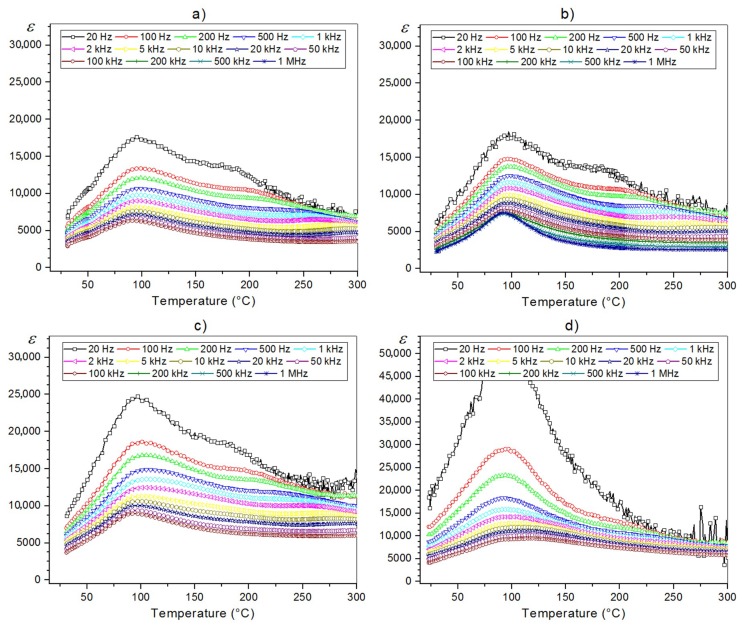
Temperature dependences of dielectric permittivity (*ε*) for the PFN ceramics: (**a**) P-1u, (**b**) P-2u, (**c**) P-3u, (**d**) P-4u.

The temperature dependences of dielectric permittivity (*ε*) for the PFN ceramic samples are presented in Figure 6. For the P-1u samples, both at room temperature and at the temperature of the phase transition with increasing sintering temperature, the values of dielectric permittivity increase. For 1 kHz and *RT* value the dielectric permittivity is 4207, 4210, and 5650, for the P-1u, P-2u, and P-3u samples, respectively. In the case of the P-4u sample, additional stages in the technological process caused an additional increase in the value of dielectric permittivity maximum (Figure 6d, Table 2). Differences in the value of the dielectric permittivity maximum of the PFN material (Table 2) resulted from different sizes and shapes of the ceramic grains of the samples. In the case of the materials with a perovskite structure, lower density and microstructure samples with poorer grain show a stronger phase transition blur.

The PFN ceramics obtained by the precipitation method (and subjected to ultrasounds) revealed a diffuse phase transition. Broadening of the phase transition is manifested by the “flattening” of the temperature dependence *ε*(*T*), i.e., the reduction in the height of the *ε_max_* (in this case the temperature corresponding to the *ε_max_* is determined by *T_m_*). Broadening of the phase transition in materials with a perovskite structure may be associated with the occurrence of local deviations from the chemical composition in the micro-areas of ceramics (in the case of the PFN ceramics, its related to disorder in the distribution of B-side ions in the perovskite unit cell), as well as a result of the heterogeneity of the distribution of defects and mechanical stresses in grains. These factors can be the corners of the formation of a new phase at temperatures quite distant from the Curie temperature (*T_C_*) of the material. The broad phase transition in the PFN ceramics is also affected by the degree of powder compaction during the technological process, as well as the thickness of ceramic samples prepared for electrical tests. Differences in the value of the dielectric permittivity maximum of the obtained PFN samples also resulted from different sizes and shapes of the ceramic grains of the samples. In the case of the ceramic materials (with a perovskite structure), samples with lower density and microstructures with poor angularity of grains show increased broadening of the phase transition.

**Figure 7:**
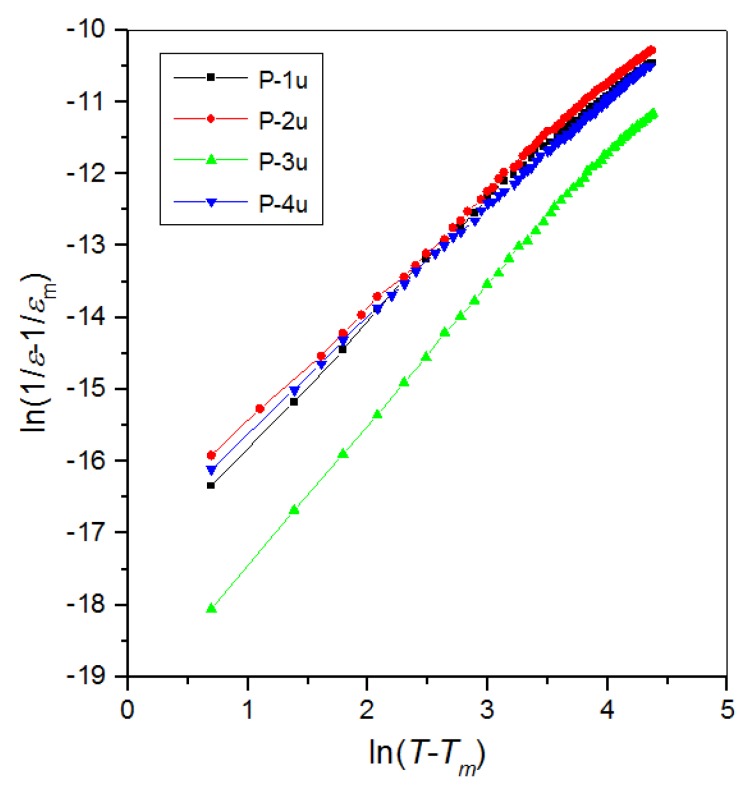
Plots of ln(1/*ε* − 1/*ε_m_*) vs. ln(*T* − *T_m_*) at temperatures higher than *T_m_* for PFN ceramics (1 kHz).

In order to evaluate the phase transition, we used the modified Curie–Weiss law (Equation (3)) [2]

(3) $$\frac{1}{\varepsilon }-\frac{1}{\varepsilon_{m}}=C{\left(T-T_{m}\right)}^{\alpha }$$

where *ε_m_* is the maximum value of dielectric constant, *T_m_* is temperature of the value dielectric permittivity maximum, *C* represents Curie–Weiss parameter, and *α* is the parameters indicating the degree of blur of the phase transition and indicated ferroelectric relaxation behavior. In this case, *α* = 1 indicates normal Curie-Weiss behavior, while *α* = 2 represents a relaxor phase transition. The *α* parameter can be calculated by the slope of the graph plotted between ln(1/*ε* − 1/*ε_m_*) and ln(*T* − *T_m_*) under 100 Hz (Figure 7) [48]. The *α* parameter calculated for the P-1u sample is 1.73, for the P-2u sample is 1.61, for the P-3u sample is 1.95, and for the P-4u sample is 1.58.

**Figure 8:**
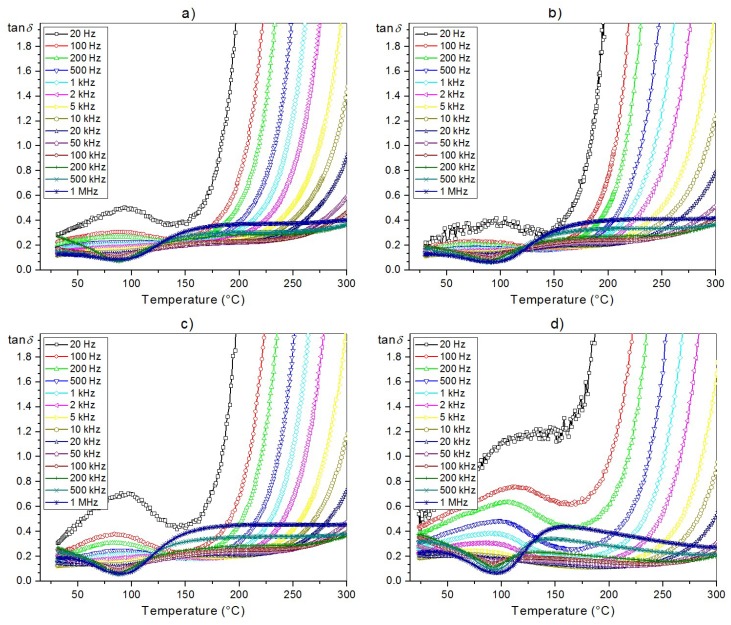
Temperature dependences of dielectric loss tangent (tan*δ*) for the PFN ceramics: (**a**) P-1u, (**b**) P-2u, (**c**) P-3u, (**d**) P-4u.

The temperature dependences of dielectric loss tangent (tan*δ*) for the ceramic PFN samples are presented in Figure 8. At room temperature *RT* and for 1 kHz, the value of tan*δ* is 0.148, 0.123, 0.136, 0.265, for the P-1u, P-2u, P-3u, and P-4u, respectively. From *RT* until about 150 °C, the dielectric loss for the PFN samples is low. Above this temperature (150 °C), a rapid growth of the dielectric loss is visible. The lowest dielectrc loss is shown in the PFN sample. The use of additional treatments in the technological process increases dielectric loss throughout the entire measurement area.

The conducted temperature tests of the dielectric properties of the ceramic PFN samples showed both high values of dielectric permittivity and low dielectric loss. This is extremely important from the point of view of designing ceramic composites with ferroic properties. Typically, in this type of ferroelectromagnetic ceramic composite, a magnetic component (for example a ferrite exhibiting much lower resistivity) will degrade the dielectric properties of the entire composite.

### 3.5. Ferroelectric Properties

**Figure 9:**
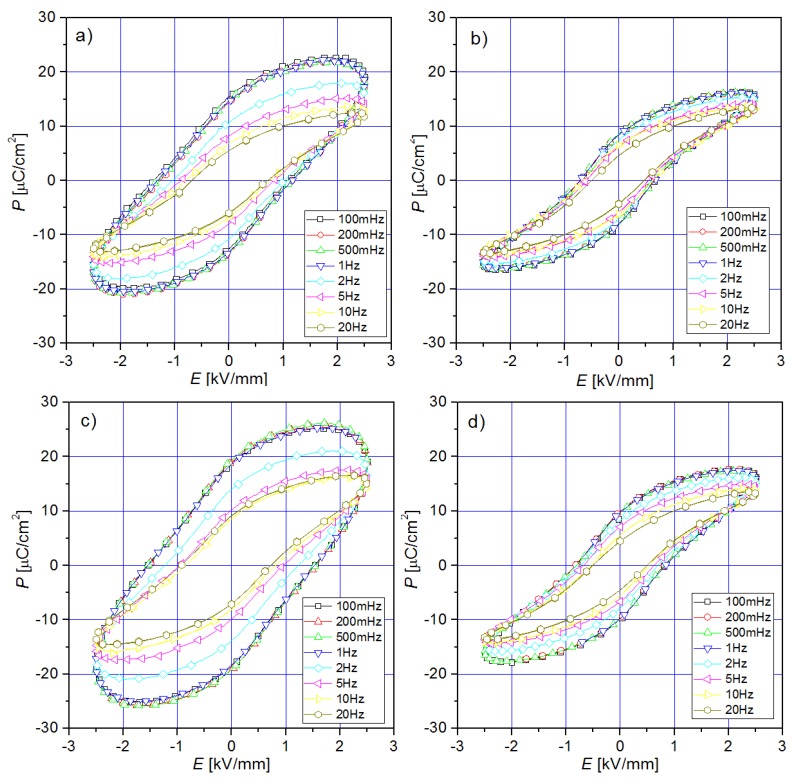
Histeresis *P*-*E* loops for PFN ceramic samples (100 mHz–20 Hz, *RT*): (**a**) P-1u, (**b**) P-2u, (**c**) P-3u, (**d**) P-4u.

The *P-E* hysteresis loops (examined at room temperature, at a frequency from 100 MHz to 20 Hz) of the PFN samples are presented in Figure 9. For all PFN samples (except for P-4u), *P-E* tests at room temperature showed that with increasing frequency, the saturation of hysteresis loops increases. In the case of P-1u and P-3u samples, at low frequencies, the loops have a characteristic shape for ceramic materials with losses. For 10 Hz, the value of remnant polarization *P_R_* is 6.76 µC/cm^2^, 5.63 µC/cm^2^ and 8.24 µC/cm^2^, and the coercive field *E_C_* is 0.785 kV/mm, 0.536 kV/mm and 0.824 kV/mm for P-1u, P-2u, and P-3u, respectively.

Despite having the lowest values for the remnant polarization and the coercive field, the hysteresis loop of the P-2u sample is narrow and shows the highest saturation for all frequencies of the measurement field. This confirms that for PFN ceramics sintering at 1050 °C is the most advantageous.

### 3.6. Magnetic Properties

**Figure 10:**
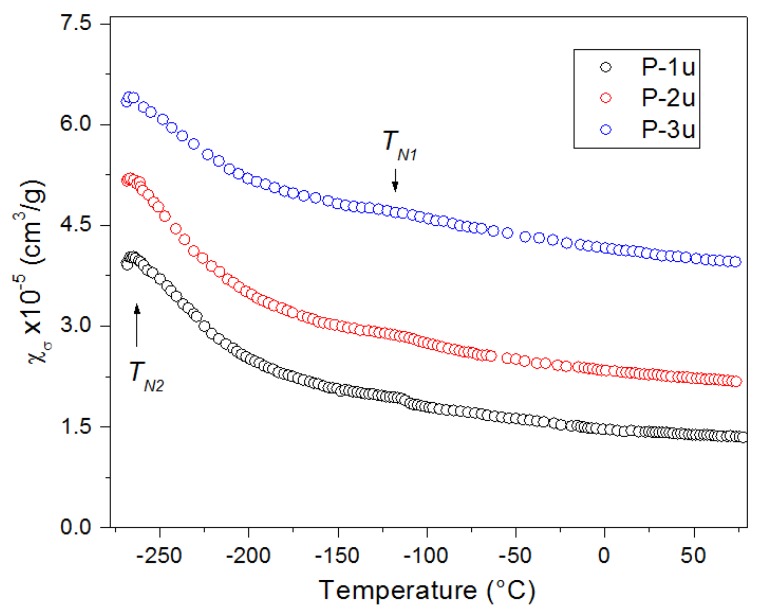
Temperature measurements of the magnetic mass susceptibility *χ_σ_* of PFN ceramic samples.

Temperature measurements of magnetic susceptibility *χ_σ_* for the PFN ceramics are presented in Figure 10. Two peaks can be observed on the temperature plots. A small peak (*T_N_*_1_) appears near the temperature of −112 °C, and a second one (*T_N_*_2_) at a temperature of about −262 °C is more visible. The anomalies appearing on the plots are connected with the occurrence of phase transitions in the magnetic material [49]. At *T_N_*_1_ (~−112 °C), the peak determines the magnetic transition (paramagnetic/antiferromagnetic), while the peak at *T_N_*_2_ (~−262 °C) is probably related to AFM-to-AFM transition [22]. The values of magnetic susceptibility decrease with an increase in the PFN ceramics’ sintering temperature, but the trend of all curves is similar. In the case of the P-4u sample, the curves are not significantly different from the P-2u sample.

## 4. Conclusions

The paper presents the chemical-wet technological process (precipitation from the solution) of ferroelectromagnetic PbFe_1/2_Nb_1/2_O_3_ ceramic powders subjected to ultrasound. The PFN material was obtained in various technological conditions and XRD, SEM, ferroelectric, dielectric, and magnetic tests were carried out. Chemically-wet methods of obtaining PFN ceramic powder have many advantages, but they also have some drawbacks. One of the disadvantages for the precipitation from the solution method is a strong tendency for the powder to combine into the form of hard agglomerates, which promotes the formation of porosity in the closed ceramic sample. Optimization of powder mixing parameters and prolonged mixing time of powders do not result in eliminating this unfavorable phenomenon. Ultrasound treatment of the PFN ceramic powder enables the partial reduction of the hard powdery agglomerates, from which the material exhibits a smaller amount of closed porosity.

At *RT*, the PFN material has shown a perovskite structure with small amount of the pyrochlore phase. The SEM microstructure analysis of the ceramic samples showed that the most correctly crystallizing grain has a sample obtained under the conditions of 1050 °C/2 h. Increasing the sintering temperature causes an increase in the average grains. At room temperature, the PFN ceramic samples show both ferroelectric and magnetic properties. Sintering temperatures that are either too low or too high deteriorate the dielectric properties of the PFN ceramic samples. The high sintering temperature (1300 °C) also contributes to excessive grain growth, as well as increasing the heterogeneity of the microstructural ceramic sample. Deprivation of homogeneity of the microstructure deteriorates the set of final electrophysical parameters of the PFN ceramic samples. Optimization of PFN technology extends the possibility of using this material to obtain multiferroic composites or multicomponent solid solutions based on PFN, for use in targeted applications in systems integrating ferroelectromagnetic properties in one device e.g., in microelectronic applications. The optimal sintering temperature for the PFN ceramic material obtained by chemical-wet technology under the previously listed conditions is 1050 °C/2 h.

## Figures and Tables

**Table 1:** Theoretical and experimental percentages of elements of the PFN ceramics (expressed as oxides).

Oxide Formula	Theoretical (%)	Experimental (%)			
		P-1u	P-2u	P-3u	P-4u
PbO	67.72	67.42	67.69	67.57	67.93
Fe_2_O_3_	12.11	11.27	11.83	12.23	12.38
Nb_2_O_5_	20.16	20.31	20.47	20.20	19.69

**Table 2:** Parameters of the PFN ceramic samples.

Parameter	P-1u	P-2u	P-3u	P-4u
*ρ* (g/cm^3^)	6.58	7.33	7.41	7.29
*T_C_* (°C) ^1^	97	97	105	97
*ε_r_* at *RT* ^1^	4207	4210	5650	7753
*ε_max_* at *T_C_* ^1^	9774	11,651	13,584	15,812
tan*δ* at *RT* ^1^	0.148	0.124	0.136	0.265
tan*δ* at *T_C_* ^1^	0.208	0.168	0.217	0.381
*ρ_DC_* at *RT* (Ωm)	8.0 × 10^6^	1.4 × 10^7^	2.4 × 10^6^	6.1 × 10^5^
*E_Act_* in I (eV)	0.454	0.489	0.451	0.430
*E_Act_* in II (eV)	0.219	0.146	0.302	0.181
*E_Act_* in III (eV)	1.034	1.121	1.015	1.067
*E_C_* (kV/mm) ^2^	0.785	0.777	0.825	0.539
*P_R_* (μC/cm^2^) ^2^	6.832	0.64	8.50	5.53

^1^ for 1 kHz, ^2^ for 10 kHz, *RT*—room temperature.
